# Fish mutant, where is thy phenotype?

**DOI:** 10.1371/journal.pgen.1007197

**Published:** 2018-02-22

**Authors:** Darius Balciunas

**Affiliations:** Department of Biology, Temple University, Philadelphia, Pennsylvania, United States of America; University of Pennsylvania School of Medicine, UNITED STATES

The field of genetics emerged as a study of the inheritance of desirable or otherwise interesting traits. Mutations were characterized as recessive or dominant, assigned to complementation groups, mapped, and finally, the affected genes were identified. By default, recessive mutations could only be isolated in genes that played an important role in the biological process of interest, be it pea color or segmentation pattern of the fruit fly embryo.

**Fig 1:**
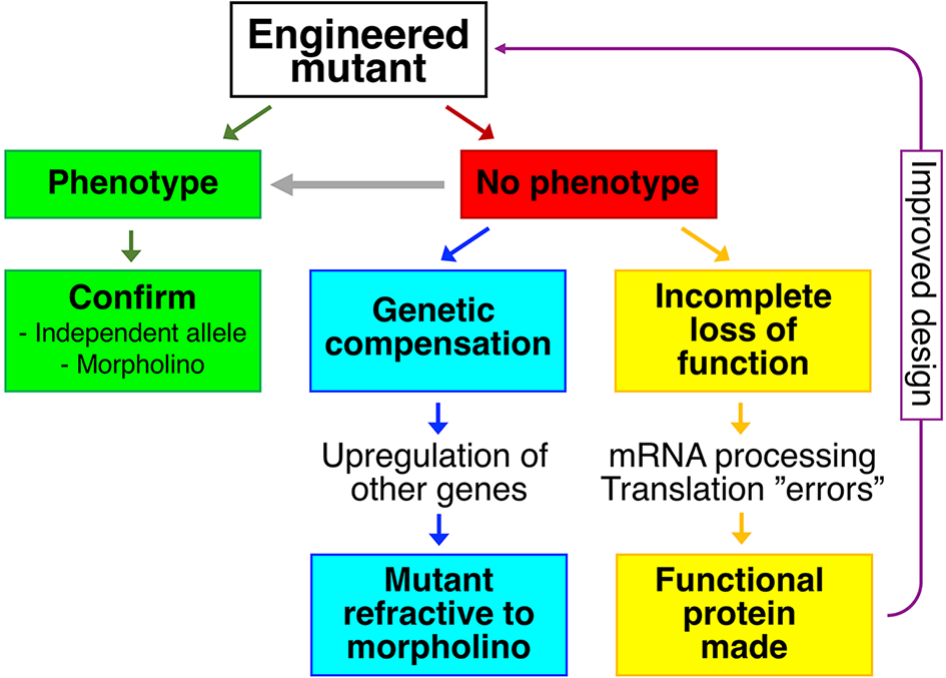
Reverse genetics and mutant phenotypes. Green panels represent a scenario where an engineered mutant displays a phenotype. In the absence of a phenotype (red panel), the possibilities of genetic compensation (blue panels) or incomplete loss of function (yellow panels) should be considered. If incomplete loss of function is likely, this information could be used to engineer a better allele (purple arrow). In mutants without overt phenotypes, more specific or more stringent assays may be needed to reveal a phenotype (grey arrow). In addition to scenarios displayed in the figure, genetic redundancy is known to lead to an absence of mutant phenotypes.

As methodologies to mutate specific genes in various model systems were developed, scientists began to employ reverse genetics, whereby a gene of interest is selected and a mutant is generated. Ideally, the mutant displays a phenotype that can be studied (green panels in Fig 1). Such best-case scenarios pose the danger of confirmation bias ([1] and references therein). It may therefore be prudent to validate the phenotype by engineering an independent mutant allele. This is especially straightforward in zebrafish, given that targeted mutagenesis using CRISPR/Cas9 requires relatively little effort [2–4]. In zebrafish, the phenotype may also be confirmed by performing knockdown using morpholino oligonucleotides [5–6].

Real experiments rarely follow best-case scenarios, and very frequently, mutants generated by reverse genetics fail to display overt phenotypes. Are the majority of protein-coding genes indeed not required, often despite a very high degree of evolutionary conservation? Genetic redundancy, most obviously in the form of homologous or duplicated genes, certainly contributes to a lack of mutant phenotypes. But are mutant phenotypes being obscured by additional mechanisms, either active or passive? Two recent publications delve into phenomena, new to the zebrafish field, that may prevent manifestation of a mutant phenotype.

The first phenomenon is genetic compensation (blue panels in Fig 1), described in zebrafish by Andrea Rossi and colleagues [7]. The hallmark of genetic compensation is up-regulation of expression of other, often closely related, genes in mutants. The proteins encoded by these other genes compensate for the loss of the protein of interest, masking the phenotype. Notably, an antisense-based morpholino knockdown case does not trigger genetic compensation [7]. Due to genetic compensation, phenotypically wild-type mutants can become refractive to morpholino-induced phenotypes, providing a critical test both for genetic compensation and for the specificity of morpholino phenotypes.

The second phenomenon is described by Jennifer Anderson and colleagues in November 2017’s issue of *PLOS Genetics* (yellow panels in Fig 1) [8]. Their paper challenges the assumption that splice site, frameshift, and nonsense mutations necessarily lead to complete loss of function (null) phenotypes. The authors clearly demonstrate that in homozygous mutants, alternative mRNA splicing and use of cryptic splice sites lead to the emergence of mRNA variants with restored open reading frames. Furthermore, they provide evidence that such mRNA variants could be translated into at least partially functional proteins. They draw a comparison to human geneticists’ discovery that normal individuals can be homozygous for apparently deleterious mutations in expected essential genes [9]. One might add that even mutant mRNAs can be translated into full-length proteins through mechanisms such as ribosomal frameshifting and nonsense readthrough. Nonsense readthrough in particular appears to be not only common but also potentially drug-targetable in humans [10].

How do these phenomena relate to a pressing issue facing the reverse genetics community (discrepancies between the phenotypes produced by antisense and genome editing approaches) [6, 11]? Genetic compensation provides one explanation, as it has been demonstrated to underlie differences between mutant and morphant phenotypes (recently reviewed in [12]). It remains to be seen how widespread the phenomenon is, but the hypothesis that genetic compensation masks a phenotype is readily testable for any zebrafish mutant/morphant pair: the morphant phenotype should be suppressed in homozygous mutants.

Incomplete loss of function, including the appearance of mRNA processing variants described by Anderson and colleagues [8], certainly has the potential to mask mutant phenotypes as well. How should one go about testing this possibility? Reading frame-restoring mRNAs can be readily detected by reverse transcription PCR and sequence analysis on homozygous mutant embryos. Translational artifacts such as nonsense readthrough are much harder to identify and even more difficult to conclusively rule out. Instead, the best practical solution may be to avoid this possibility altogether by engineering the mutants to be nulls in the first place. Anderson and colleagues provide excellent common-sense guidelines for improved mutant design, including targeting of protein domains known to be essential for function and avoiding exons which begin and end in the same phase.

It also remains to be seen if a significant fraction of mutant/morphant phenotype discrepancies will be explained solely by incomplete loss-of-function mutants. Even when frameshift, nonsense, or splice site mutants are not complete nulls, only a small amount of the active protein will be made in most cases. Likewise, morpholinos usually do not completely shut down the expression of the target genes, also leading to a small amount of wild-type protein being made. Then why would a mutant have a less severe phenotype than a morphant? At least two straightforward yet opposing explanations exist. One is that a larger amount of target protein is made in the mutant compared to morphant, which would have to be determined on a case-by-case basis. The other explanation is that morphant phenotypes are due to well-documented off-target effects of morpholino oligonucleotides, requiring careful reassessment of knockdown experiments [6].

The main underlying question—why do the majority of genes not yield an overt phenotype when mutated?—is still open. How do such “unnecessary” genes remain evolutionarily conserved? The simplest answer comes back to thorough phenotyping (grey arrow in Fig 1). For example, mutants in genes regulating subtle metabolic adaptations or behaviors are likely to appear “normal” unless specific assays are performed. Second, the highly controlled conditions under which we raise and maintain our fish are a far cry from the constantly changing natural environment full of predators and parasites. Finally, the phenotype does not need to be all-or-nothing. In *Saccharomyces cerevisiae*, fewer than 20% of genes are defined as “essential.” A much larger fraction of mutants yields a measurable effect on relative fitness when tested in competitive culture assays [13]. Similar multigenerational studies are of course not feasible for zebrafish. Yet a fitness effect of a few percentage points may be more than enough to provide purifying selection, resulting in strong sequence conservation.
